# Influence of cytokines on early death and coagulopathy in newly diagnosed patients with acute promyelocytic leukemia

**DOI:** 10.3389/fimmu.2023.1100151

**Published:** 2023-03-31

**Authors:** Shixiang Zhao, Yuanyuan Ge, Zengzheng Li, Tonghua Yang

**Affiliations:** ^1^ Faculty of Life Science and Technology, Kunming University of Science and Technology, Kunming, China; ^2^ Department of Hematology, The First People’s Hospital of Yunnan Province, Kunming, China; ^3^ Yunnan Blood Disease Clinical Medical Center, The First People’s Hospital of Yunnan Province, Kunming, China; ^4^ Yunnan Blood Disease Hospital, The First People’s Hospital of Yunnan Province, Kunming, China; ^5^ Department of Cardiology, 920th Hospital of Joint Logistics Support Force, Kunming, China

**Keywords:** acute promyelocytic leukemia (APL), early death (ED), coagulopathy, intracranial hemorrhage (ICH), interleukin-17A (IL-17A)

## Abstract

**Introduction:**

Acute promyelocytic leukemia (APL) is a subtype of acute myeloid leukemia (AML) with a better prognosis. But early death (ED) rate remains high. APL patients are simultaneously accompanied by coagulopathy and hyperinflammation at the onset. It is not known what effects cytokines have on ED and coagulopathy in these patients. Therefore, the purposes of this study are to explore the clinical differences between APL and other types of AML, the link between cytokines and coagulopathy in newly diagnosed APL, and their roles in the ED for APL.

**Methods:**

This study retrospectively collected the information of 496 adult patients with AML (age ≥14 years at admission) newly diagnosed in the First People's Hospital of Yunnan Province between January 2017 to February 2022, including 115 APL patients. The difference of clinical manifestations between two groups [APL and AML (non-APL)] was statistically analyzed. Then, the factors affecting ED in APL patients were screened, and the possible pathways of their influence on ED were further analyzed.

**Results:**

The results indicate APL at the onset have a younger age and higher incidence of ED and DIC than other types of AML. Intracranial hemorrhage (ICH), age, and PLT count are found to be independent factors for ED in newly APL, among which ICH is the main cause of ED, accounting for 61.54% (8/13). The levels of cytokines in newly APL are generally higher than that in AML (non-APL), and those in the group of ED for APL were widely more than the control group. IL-17A and TNF-β are directly related to the ED in newly APL, especially IL-17A, which also affects ICH in these patients. Moreover, the increase of IL-17A and TNF-β cause the prolongation of PT in APL patients, which reflected the exogenous coagulation pathway. However, they have no effect on APTT prolongation and FIB reduction. Thus, it is speculated that IL-17A leads to early cerebral hemorrhage death in newly APL by inducing tissue factor (TF) overexpression to initiate exogenous coagulation and further leading to excessive depletion of clotting factors and prolongation of PT.

**Conclusions:**

In conclusion, compared with other types of AML, APL patients have a younger age of onset and high inflammatory state, and are more likely to develop into DIC and die early. Age, and PLT count at diagnosis are independent factors for ED of APL, especially ICH. IL-17A is confirmed to be an independent risk factor for ED and ICH of newly APL. Hence, IL-17A may serve as a predictor of ED in newly diagnosed APL patients, and controlling its expression probably reduce ED in these patients.

## Introduction

Acute promyelocytic leukemia (APL) with t(15;17)(q22; q12) is a distinct subtype of acute myeloid leukemia (AML), accounting for 5%-20% of AML cases (1–4). Although APL was previously considered one of the most rapidly lethal forms of AML (3), the recent application of the target-specific agents all-trans retinoic acid (ATRA) and arsenic trioxide (ATO), together with improvements in supportive treatment, has improved the long-term survival of APL patients considerably. Nevertheless, early mortality (defined as death within 0 to 30 days of diagnosis) still remains high in multiple APL cohorts, accounting for 8.2-32.6% of overall mortality, and early death (ED) is the greatest contributor to treatment failure in APL patients (5–11).

ED for APL is frequently associated with the presenting coagulopathy. In a registry study of 105 APL patients, early mortality was reported to be 29%, with hemorrhage being the most common cause of death, accounting for 41% of ED patients, with the early death rate (EDR) also increasing with age (8). Another study involving newly diagnosed APL patients found an EDR of 11%, with hemorrhage as the cause of early death in 61% of cases (12). Park JH, et al. reported an EDR of 17.3% as a result of bleeding despite the use of ATRA (10). To improve the prognosis of patients with APL, more attention needs to be paid to the persistently high EDR in patients with newly diagnosed APL, the main cause of which is life-threatening coagulopathy and fatal hemorrhage (13).

APL is a hyperacute disease and often presents with disseminated intravascular coagulation (DIC) with secondary bleeding. A study of 116 APL patients reported an incidence of overt DIC of 77.6% with prolonged prothrombin time (PT) being a risk factor for bleeding (14). Furthermore, APL is usually accompanied by elevated levels of multiple cytokines leading to leukocyte proliferation after treatment with ATRA or ATO (15). Both the differentiation syndrome (DS) and cytokine storm have been frequently associated with APL onset or induction therapy (16). The National Comprehensive Cancer Network (NCCN) guidelines recommend that high-risk APL patients with white blood cell (WBC) counts over 10×10^9^/L be treated with corticosteroids, which can correct existing or worsening coagulopathy (17).

Although it is known that APL in patients is often accompanied by DIC and hyperinflammation at onset, the link between them and the roles they play in early APL-related death remain unclear. APL is known to be a specific subtype of AML, but the differences in the clinical manifestations between APL and other subtypes of AML at the initial stages are not fully understood. Thus, the objectives of this study were to evaluate the differences in clinical presentations between APL and other types of AML in patients and to further identify the cytokines that influence APL-related ED, as well as other influencing factors.

## Materials and methods

### Study population

A total of 496 patients with newly diagnosed adult AML (age ≥14 years at the time of admission) were enrolled in the cohort between January 2017 and February 2022; these included 115 APL patients. Fasting venous blood was collected from all subjects on the day or day following admission. The data compiled at admission (i.e., not receiving ATRA, ATO, or chemotherapy, at our or another hospital) were used for analysis. Patients who were not newly diagnosed or lacked initial data were excluded from the cohort. The general characteristics of these patients and their laboratory data are shown in **Table 1**.

**Table 1:** Comparison of clinical characteristics between newly diagnosed APL and AML (non-APL) patients.

Characteristics	APL (n=115)	AML (non-APL) (n=381)	Statistics	*P*
Sex *M/F*	53/62	193/188	*χ^2 =^* 0.738	0.390
Age(years) *M(IQR)*	38(26-53)	53(36-63)	*Z*=-5.568	0.000^**^
LDH(U/L) *M(IQR)*	305(205-493)	363(229-644)	*Z*=-1.917	0.055
AST(U/L) *M(IQR)*	24(18-32)	17(11-27)	*Z*=-4.967	0.000^**^
ALT(U/L) *M(IQR)*	19.3(13.5-38)	19(14-27)	*Z*=-1.052	0.293
WBC(×10^9^/L) *M(IQR)*	2.41(1.08-9.91)	11.26(2.96-42.55)	*Z*=-6.448	0.000^**^
HGB(g/L) *M(IQR)*	83(71-100)	78(67-93)	*Z*=-2.558	0.011^*^
PLT(×10^9^/L) *M(IQR)*	23(13.5-39)	39(22-77)	*Z*=-5.636	0.000^**^
Ferritin (ng/ml) *M(IQR)*	850.54(430.47-1711.15)	930.88(471.66-1863.59)	*Z*=-0.814	0.416
Tumor cells (%) *M(IQR)*	86.5(82-91.5)	66.5(40.5-82.5)	*Z*=-9.281	0.000^**^
Fever	54(46.96%)	146(38.32%)	*χ^2 =^* 2.738	0.098
Fatigue	51(44.35%)	218(57.22%)	*χ^2 =^* 5.895	0.015^*^
Splenomegaly	15(13.04%)	72(18.90%)	*χ^2 =^* 2.093	0.148
Bleeding	99(86.09%)	138(36.22%)	*χ^2 =^* 88.039	0.000^**^
ICH	12(10.43%)	13(3.41%)	*χ^2 =^* 9.102	0.003^**^
Early death	13(11.30%)	11(2.89%)	*χ^2 =^* 13.592	0.000^**^

F, female; M, male; LDH, lactate dehydrogenase; AST, aspartate aminotransferase; ALT, alanine aminotransferase; WBC, white blood cell; HGB, hemoglobin; PTL, platelet; ICH, Intracranial hemorrhage; Fever, body temperature over 37.4°C; *P< 0.05, **P< 0.01.

All patients with suspected APL were started immediately on oral ATRA (25 mg/m^2^/day). After confirmation of the APL diagnosis, ATO (0.16 mg/kg/day) combined with ATRA was administered as dual-induction therapy until complete remission. During induction therapy, chemotherapy was administered when the WBC count was higher than 4×10^9^/L. The dosage of the combined chemotherapy agents (hydroxyurea [3.0 g/day]), daunorubicin [25–45 mg/m^2^/day], idarubicin [8–12 mg/m^2^/day], or cytarabine [100 mg/m^2^/day] was given according to the Chinese Diagnosis and Treatment Guidelines for APL (2018, 2021 version). Of course, dosage and duration should be adjusted appropriately according to the patient’s WBC count, tolerance, and complications.

Sites of bleeding include the skin, mucous membranes (nasal cavity, oral cavity, and bulbar conjunctiva), gums, digestive tract, muscles, uterus, and intracranial hemorrhage (ICH). The presence of ICH was assessed by clinical manifestations and was further confirmed by computed tomography (CT) or magnetic resonance imaging (MRI). DIC scores were determined according to the SSC/ISTH (scores ≥5) (18) and CDSS (scores ≥6) (19) scoring systems. This study strictly followed the guidelines of the Helsinki Declaration and the International Code of Ethics for Biomedical Research Involving Humans, jointly formulated by the World Health Organization and the Council of International Medical Science Organization, as well as the relevant regulations of the Ethics Committee of the First People’s Hospital of Yunnan Province. All participants provided written informed consent.

### Cytokine determination

Fasting venous blood was collected in serum separator tubes (Jiamay Biotech, Beijing, China) to obtain serum for the detection of cytokine concentrations. According to the manufacturer’s instructions, the serum levels of interferon (IFN) -γ, interleukin (IL)-1β, IL-2, IL-4, IL-5, IL-6, IL-8, IL-10, IL-12p70, IL-17A, IL-17F, IL-22, tumor necrosis factor (TNF)-α and TNF-β were quantified using an Aimplex Cytokine kit (QuantoBio, Tianjing, China) with the detection range of 2.3-5000 pg/mL. Flow cytometry was performed using a NovoCyte D2060R instrument that was purchased from ACEA Biosciences Inc. (San Diego, CA, USA).

### Statistical analysis

The distribution of the data was tested using the Kolmogorov-Smirnov (samples greater than 50) or Shapiro-Wilk (samples less than 50) tests. Data conforming to the normal distribution were expressed as means ± standard deviation (*M ± SD*) and compared using the t-test. Non-normally distributed data were expressed as medians with interquartile ranges (*M[IQR])* and compared with the nonparametric Mann-Whitney U test. The *χ^2^* test was used to compare categorical variables between groups. Binary logistic regression was used to identify candidate variables with significant (*P*< 0.05) influence on ED and ICH, using univariate analysis followed by multivariate analysis. Non-parametric Spearman’s product-moment correlation analysis was used to examine the relationship between leukocyte counts at diagnosis and risks affecting ED. SPSS 21.0 (IBM Corp., Armonk, NY, USA) was used for statistical calculation, and GraphPad Prism 8 (San Diego, CA, USA) was used to construct the forest map. Optimal cutoff values of parameters were determined by receiver operating characteristic (ROC) curves. All tests were two-tailed and *P*< 0.05 was considered statistically significant.

## Results

### Comparison of characteristics between APL and AML (non-APL) patients

APL is an exceptional AML subtype, characterized by the t(15;17)(q22; q12) chromosomal rearrangement, creating the *PML-RARA* fusion gene. Its early high mortality and excellent prognosis differ from those of AML. **Table 1** summarizes the clinical characteristics of the 496 newly diagnosed adult AML patients (APL=115, AML [non-APL] =381). The incidence was similar between men and women in both groups. The APL patients were found to be significantly younger than other AML patients at diagnosis, with a median age of 38 (26–53) years and 53 (36–63) years at the time of diagnosis, respectively (*P*< 0.01). In terms of the peripheral blood counts, both the WBC and platelet (PLT) counts were lower in patients with APL than in non-APL patients (WBC 2.41 [1.08-9.91] *vs* 11.26 [2.96-42.55] ×10^9^/L, PLT 23 [13.5-39] *vs* 39 [22-77] ×10^9^/L) (*P*< 0.01), while the level of hemoglobin (HGB) was higher than that in the control group [83 [71-100] *vs* 78 [67-93] g/L) (*P*< 0.05). Regarding other blood tests, aspartate aminotransferase (AST) was higher in APL patients than in the control patients (24[18-32] *vs* 17[11-27] U/L) (*P*< 0.01), while no differences were seen in the levels of alanine aminotransferase (ALT), lactate dehydrogenase (LDH), and ferritin between the two groups (*P*> 0.05). There was no significant difference in the proportions of splenomegaly and fever between the two groups, although APL patients were more prone to fatigue (*P*< 0.05) and bleeding (*P*< 0.01), and the proportion of tumor cells in the bone marrow was higher than that in other AML patients (86.5[82-91.5] *vs* 66.5[40.5-82.5] %) (*P*< 0.01). Compared with other AML patients, those with APL had a higher incidence of ICH (10.43 *vs* 3.41%) and ED (11.30 *vs* 2.89%) (*P*< 0.01) (**Table 1**). Furthermore, the median time of ED (time from first admission to death) was 4 (2-4.5) days in APL and 5 (3–22) days in AML without APL. The APL patients were then divided into three risk groups according to the PETHEMA/GIMEMA risk classification (20). The numerical distribution of the patients in the three groups was low-risk 21 (18.26%), intermediate-risk 65 (56.52%), and high-risk 29 (25.22%) in this study. The ED rates were 9.3% (8/86) in the low/intermediate-risk group and 17.24% (5/29) in the high-risk group.

### Factors influencing early death in APL patients

**Figure 1:**
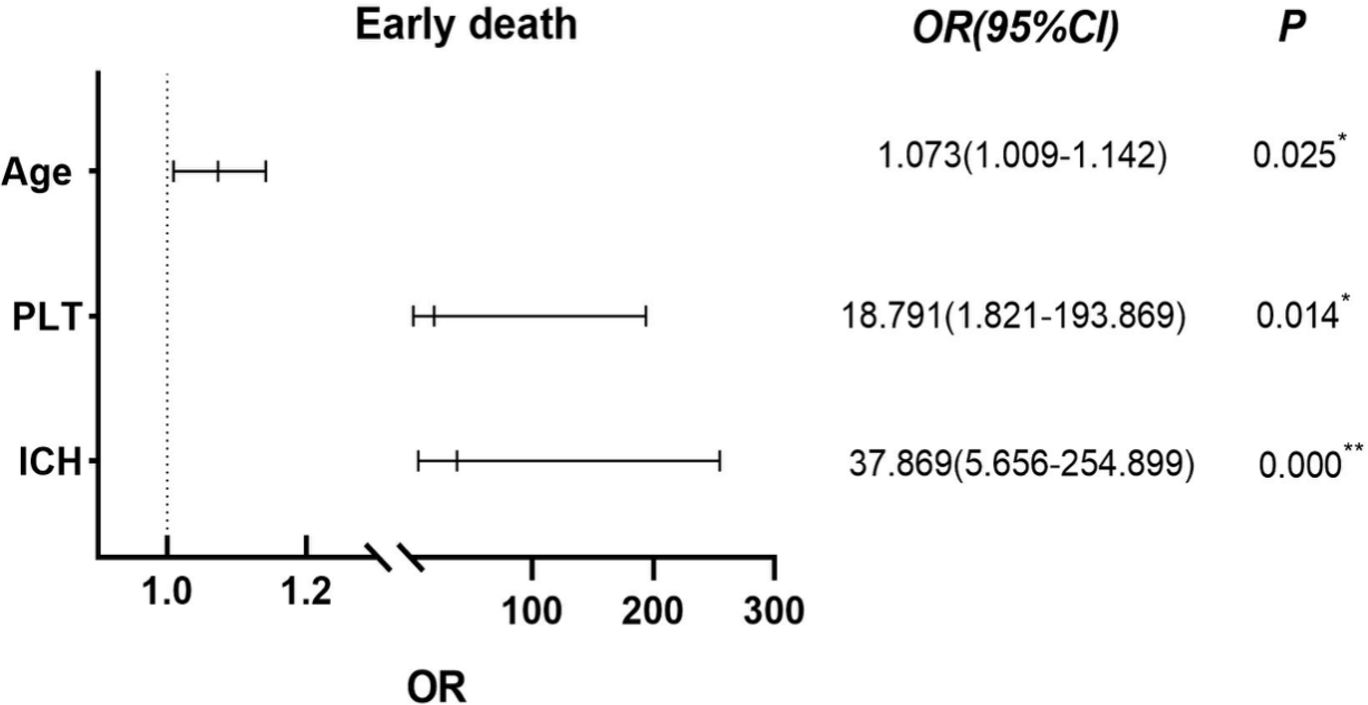
Using Binary Logistic Regression, statistically significant variables were screened out by univariate analysis and further multivariate analysis was used to test showed that age, ICH and PLT below 20 ×10^9^/L were independent risk factors for ED in APL patients. *P< 0.05, **P< 0.01.

The 13 early deaths (defined as death within 0 to 30 days of diagnosis) that occurred in the 115 APL patients were primarily caused by intracranial hemorrhage (n=8, 61.54%), severe pulmonary infection combined with respiratory failure (n=3, 23.08%), sepsis with acute heart failure (n=1, 7.69%), and tumor lysis syndrome with multiorgan failure (n=1, 7.69%). The clinical factors shown in **Table 1**, especially the parameters with statistical differences, were included in the univariate analysis to determine their effects on ED in APL patients, together with other coagulation indicators such as PT, activated partial thromboplastin time (APTT), D-dimer (D-Di), fibrinogen (FIB), and fibrinogen degradation products (FDP). The results indicated that increased age, prolonged PT, ICH, PLT counts less than 20×10^9^/L, and elevated LDH levels differed significantly between ED and non-ED patients in the univariate analysis (*P*< 0.05), while the multivariate analysis showed significantly increased risks of ED associated with the odds ratios (*OR*) of ICH (37.869 [95% confidence interval {*CI*}, 5.656 to 254.899), PLT counts below 20×10^9^/L (18.791 [95%*CI*, 1.821 to 193.869]), and age (1.071 [95%*CI*, 1.009 to 1.142]). PLT counts and age were significant at the *P*< 0.05 level while ICH was significant at the *P*< 0.01 level (**Figure 1**). Spearman’s product-moment correlation analysis was further conducted for risk factors associated with ED identified by the univariate analysis, showing that WBC counts at diagnosis were directly correlated with LDH concentration (*r*= 0.670, *P*< 0.01) and PT time (*r*= 0.291, *P*< 0.01), but showed no correlation with PLT counts or age (*P*> 0.05). In addition, the WBC counts at diagnosis affected ICH in APL patients (*OR*=1.021, 95% *CI*=1.006-1.037) (*P*< 0.01). Other clinical parameters such as sex, WBC counts themselves, AST, ALT, fever, bleeding, bone marrow tumor cells, and splenomegaly, had no significant effects on ED. Of the 13 APL patients who experienced ED, eight (61.54%) died from ICH, indicating the importance of ICH as a risk factor for ED. The incidence of ED and ICH in newly diagnosed APL was higher than that in newly AML excluding APL. At the same time, ICH accounted for the highest proportion of ED (8/13, 61.54%) in APL patients. Thus, there was a definite correlation between cerebral hemorrhage and ED. The incidence rate of DIC in APL was more than that in AML (non-APL) (82.61 *vs* 16.01%) and various coagulation indices were also worse than in other AML patients, except for APTT (*P*< 0.01) (**Table 2**), while APTT values for both APL and AML (non-APL) were within the normal reference range (range from 28 to 43.5 seconds).

**Table 2:** Comparison of coagulation function between newly diagnosed APL and AML (non-APL) patients.

Characteristics	APL (n=115)	AML (n=381)	Statistics	*P*
DIC	95(82.61%)	61(16.01%)	*χ^2 =^* 181.73	0.000^**^
APTT(s) *M(IQR)*	36.2(33.65-38.2)	36.9(33.90-40.75)	*Z*=-2.254	0.024^*^
PT(s) *M(IQR)*	15.8(14.45-17.4)	14.1(13.2-15)	*Z*=-8.124	0.000^**^
FIB(g/L) *M(IQR)*	1.57(1.07-2.29)	3.98(3.14-5.03)	*Z*=-12.604	0.000^**^
FDP(ug/ml) *M(IQR)*	82.7(47.1-123)	6.1(3.1-17.2)	*Z*=-12.237	0.000^**^
D-Di(ug/ml) *M(IQR)*	29.75(14.1-49.48)	2.87(1.6-8.6)	*Z*=-11.570	0.000^**^

DIC, Diffuse intravascular coagulation; APTT, activated partial thromboplastin time; PT, prothrombin time; (s), second; FIB, fibrinogen; FDP, Fibrinogen degradation products; D-Di, D dimer. *P< 0.05, **P< 0.01.

### Comparison of cytokines between newly diagnosed APL and AML (non-APL) patients

The levels of following 11 cytokines measured in the APL patients were generally higher than in patients with other types of AML: IL-1β 1.94 (1.22-2.93) *vs* 1.62 (1.09-2.27) pg/ml, IL-5 2.17 (1.24-3.23) *vs* 1.82 (1.14-2.59) pg/ml, IL-6 9.61 (5.05-33.76) *vs* 7.98(3.25-15.05) pg/ml, IL-8 19.93 (8.86-37.49) *vs* 13.81 (6.32-35.23) pg/ml, IL-10 4.81 (3.43-6.86) *vs* 4.26(2.99-5.99) pg/ml, IL-12p70 4.02 (3.18-5.37) *vs* 3.62 (2.74-4.66) pg/ml, IL-17A 2.63 (1.46-4.18) *vs* 2.06 (1.26-3.17) pg/ml, IL-17F 3.14 (1.78-4.89) *vs* 2.57 (1.4-4.06) pg/ml, IFN-γ 1.34 (0.61-2.10) *vs* 1.18 (0.48-1.74) pg/ml, IL-22 1.34 (0.61-2.10) *vs* 1.18 (0.48-1.74) pg/ml, TNF-β 2.68 (1.65-4.9) *vs* 1.95 (1.10-3.40) pg/ml (*P*< 0.05), except for IL-2, IL-4 and TNF-α (*P*> 0.05) (**Table 3**; **Supplementary Figure 1**), indicating that newly diagnosed APL patients had high levels of inflammation. In addition, coagulation disorders and ED are known to be more severe in APL than in AML. Thus, it is speculated that cytokines may be involved in coagulation dysfunctions and ED in APL.

**Table 3:** Comparison of cytokine levels between newly diagnosed APL and AML (non-APL) patients (pg/ml, *M[IQR]*).

Cytokines	Serum Concentration in APL patients (n=115)	Serum Concentration in AML patients (n=381)	Statistics	*P*
IL-1β	1.94(1.22-2.93)	1.62(1.09-2.27)	*Z*=-2.474	0.013^*^
IL-2	2.9(1.78-4.39)	3.07(1.86-4.22)	*Z*=-0.664	0.507
IL-4	2.58(1.20-3.89)	2.60(1.51-4.02)	*Z*=-0.878	0.380
IL-5	2.17(1.24-3.23)	1.82(1.14-2.59)	*Z*=-2.289	0.022^*^
IL-6	9.61(5.05-33.76)	7.98(3.25-15.05)	*Z*=-2.937	0.003^**^
IL-8	19.93(8.86-37.49)	13.81(6.32-35.23)	*Z*=-2.318	0.020^*^
IL-10	4.81(3.43-6.86)	4.26(2.99-5.99)	*Z*=-2.087	0.037^*^
IL-12p70	4.02(3.18-5.37)	3.62(2.74-4.66)	*Z*=-2.523	0.012^*^
IL-17A	2.63(1.46-4.18)	2.06(1.26-3.17)	*Z*=-2.784	0.005^**^
IL-17F	3.14(1.78-4.89)	2.57(1.4-4.06)	*Z*=-2.432	0.015^*^
IL-22	1.34(0.61-2.10)	1.18(0.48-1.74)	*Z*=-2.079	0.038^*^
IFN-γ	2.68(1.65-4.9)	1.95(1.10-3.40)	*Z*=-3.901	0.000^**^
TNF-α	2.77(1.40-3.58)	2.81(1.69-4.01)	*Z*=-1.424	0.154
TNF-β	3.12(1.41-4.01)	2.55(1.42-3.46)	*Z*=-2.378	0.017^*^

*P< 0.05, **P< 0.01.

### Effect of cytokines on coagulation function and early death in newly diagnosed APL patients

The expression levels of five cytokines in APL patients with normal PT or prolonged PT were IL-1β 1.78 (2.74-1.2) *vs* 2.57 (4.91-1.47) pg/ml, IL-10 4.35 (6.31-3.13) *vs* 6.98 (31.44-5.07) pg/ml, IL-17A 2.34 (3.87-1.3) *vs* 3.59 (12.74-2.77) pg/ml, IL-17F 2.95 (4.42-1.72) *vs* 4.47 (10.49-3.16) pg/ml, TNF-β 2.87 (3.55-1.21) *vs* 10.12 (15.1-3.57) pg/ml (*P*< 0.05), suggesting that these cytokines may influence PT elongation. However, the levels of the remaining 10 cytokines examined did not differ significantly between the two groups (*P*> 0.05). Meanwhile, none of the cytokines evaluated in this cohort had a statistically significant effect on APTT prolongation and FIB reduction (*P*> 0.05) (**Table 4** and **Supplementary Figure 2**).

**Table 4:** Impact of cytokine levels on blood coagulation in newly diagnosed APL patients (pg/ml, *M[IQR])*.

Cytokines	APTT		*P*	PT		*P*	FIB		P
	Normal	Prolonged		Normal	Prolonged		Normal	Reduced	
IL-1β	1.87(1.22-2.92)	2.52(1.47-2.93)	0.317	1.78(1.2-2.74)	2.57(1.47-4.91)	0.020^*^	1.87(1.28-2.22)	1.96(1.18-3.17)	0.363
IL-2	2.91(1.77-4.34)	2.87(2.09-4.9)	0.606	2.77(1.67-4.18)	3.37(2.42-6.01)	0.084	2.77(1.77-3.65)	2.98(1.82-4.61)	0.398
IL-4	2.45(1.08-3.94)	3.3(2.69-3.58)	0.198	2.58(1.13-3.78)	2.13(1.24-5.34)	0.921	2.48(1.79-3.52)	2.63(1.02-4.38)	0.718
IL-5	2.29(1.26-3.23)	1.68(1.16-2.68)	0.662	2.06(1.23-3.16)	2.69(1.26-8.4)	0.119	1.92(1.26-3.05)	2.29(1.23-3.5)	0.451
IL-6	9.73(5.16-31.5)	6.71(3.9-36.22)	0.795	9.5(4.8-25.4)	14.77(5.9-73.16)	0.219	13.02(6.71-26.52)	8.45(4.65-21.89)	0.117
IL-8	18.25(8.42-37.35)	29.06(25.66-38.87)	0.160	19.93(8.86-35.2)	20.9(9.19-74.15)	0.495	25.06(11.67-45.9)	18.71(8.39-33.64)	0.299
IL-10	4.8(3.45-6.74)	5.43(3.2-8.19)	0.677	4.35(3.13-6.31)	6.98(5.07-31.44)	0.001^**^	4.26(3.15-5.66)	5.32(3.45-9.26)	0.162
IL-12p70	4.05(3.19-5.39)	3.55(2.85-3.9)	0.252	3.92(3.17-5.26)	4.22(3.45-5.57)	0.276	4.07(3.45-5.24)	3.93(3.15-5.5)	0.630
IL-17A	2.53(1.45-4.21)	2.97(2.43-3.79)	0.359	2.34(1.3-3.87)	3.59(2.77-12.74)	0.005^**^	2.32(1.3-3.69)	2.76(1.52-4.35)	0.252
IL-17F	3.27(1.79-4.92)	2.07(0.56-3.1)	0.110	2.95(1.72-4.42)	4.47(3.16-10.49)	0.011*^*^	3.09(2.02-4.15)	3.14(1.77-5.17)	0.399
IL-22	1.33(0.59-2.12)	1.39(0.65-1.58)	0.783	1.25(0.61-2.05)	1.61(0.6-2.97)	0.349	1.38(0.81-1.84)	1.25(0.59-2.33)	0.993
IFN-γ	2.68(1.67-4.85)	2.85(1.3-5.43)	0.835	2.78(1.72-4.84)	2.58(1.5-5.87)	0.629	2.93(1.69-4.56)	2.55(1.63-5.07)	0.816
TNF-α	2.80(1.41-3.59)	2.71(1.31-3.21)	0.819	2.69(1.27-3.52)	3.02(2.54-4.04)	0.172	2.65(1.65-3.4)	2.89(1.34-3.95)	0.708
TNF-β	3.14(1.41-3.97)	3.1(1.3-4.19)	0.847	2.87(1.21-3.55)	10.12(3.57-15.1)	0.000*^*^	2.84(0.84-3.55)	3.23(1.68-4.35)	0.077

The level of APTT prolongation was over than 43.5s; The level of PT prolongation was over than 18s; The level of FIB reduction was lower than 2g/L. *P< 0.05, **P< 0.01.

The expression levels of cytokines in newly diagnosed APL patients with ED were IL-1β 5.56 (2.19-6.61) pg/ml, IL-5 10.40 (3.19-15.7) pg/ml, IL-6 69.31 (12.24-84.37) pg/ml, IL-8 65.96 (11.42-290.65) pg/ml, IL-10 29.26 (4.35-55.26) pg/ml, IL-12p70 5.24 (3.93-7.98) pg/ml, IL-17A 12.76 (9.94-14.55) pg/ml, IL-17F 9.26 (3.70-13.76) pg/ml, IL-22 2.12 (1.49-3.79) pg/ml, and TNF-β 11.66 (8.84-15.46) pg/ml. The cytokine levels in survivors were IL-1β 1.75 (1.18-2.61) pg/ml, IL-5 1.92 (1.20-3.08) pg/ml, IL-6 8.90 (4.93-21.53) pg/ml, IL-8 18.25 (8.62-33.64) pg/ml, IL-10 4.67 (3.40-6.36) pg/ml, IL-12p70 3.92(3.17-5.12) pg/ml, IL-17A 2.38 (1.27-3.46) pg/ml, IL-17F 3.05 (1.67-4.25) pg/ml, IL-22 1.25 (0.95-1.97) pg/ml, and TNF-β 2.90 (1.31-3.61) pg/ml. The nonparametric Mann-Whitney U test showed that the levels of these cytokines at diagnosis were significantly higher in APL with ED than in APL survivors (*P*< 0.05), except for IL-2, IL-4, IFN-γ, and TNF-α (*P*> 0.05) (**Table 5** and **Supplementary Figure 3**).

**Table 5:** Effects of cytokine levels on early death (ED) and differentiation syndrome (DS) in newly diagnosed APL patients (pg/ml, *M[IQR]*).

Cytokines	Serum Concentration in APL patients with or without ED		*P*	Serum Concentration in APL patients with or without DS		P
	No (n=102)	Yes (n=13)		No (n=86)	Yes (n=29)	
IL-1β	1.75(1.18-2.61)	5.56(2.19-6.61)	0.000^**^	1.81(1.08-2.64)	2.09(1.45-3.82)	0.044^*^
IL-2	2.89(1.69-4.26)	3.43(2.39-9.61)	0.109	2.77(1.71-4.37)	3.22(2.39-5.42)	0.151
IL-4	2.45(1.08-3.58)	3.84(1.29-8.81)	0.081	2.11(1.03-3.51)	3.23(2.27-4.57)	0.018^*^
IL-5	1.92(1.20-3.08)	10.40(3.19-15.7)	0.000^**^	1.85(1.17-3.03)	3.08(1.91-4.04)	0.003^**^
IL-6	8.90(4.93-21.53)	69.31(12.24-84.37)	0.025^*^	9.78(4.78-30.93)	9.53(5.27-21.89)	0.812
IL-8	18.25(8.62-33.64)	65.96(11.42-290.65)	0.021^*^	20.2(8.78-37.35)	16.46(9.09-37.35)	0.812
IL-10	4.67(3.40-6.36)	29.26(4.35-55.26)	0.007^**^	4.35(3.25-6.48)	6.67(4.53-23.43)	0.016^*^
IL-12p70	3.92(3.17-5.12)	5.24(3.93-7.98)	0.021^*^	3.93(3.11-5.22)	4.42(3.36-6.19)	0.084
IL-17A	2.38(1.27-3.46)	12.76(9.94-14.55)	0.000^**^	2.35(1.34-3.45)	3.79(1.66-8.80)	0.008^**^
IL-17F	3.05(1.67-4.25)	9.26(3.70-13.76)	0.002^**^	3.03(1.66-4.83)	3.39(2.5-5.10)	0.301
IL-22	1.25(0.95-1.97)	2.12(1.49-3.79)	0.042^*^	1.23(0.52-2.01)	1.55(1.01-2.60)	0.066
IFN-γ	2.68(1.67-4.76)	2.90(1.61-7.30)	0.336	2.66(1.62-4.62)	2.90(2.05-5.74)	0.265
TNF-α	2.74(1.31-3.47)	3.00(1.76-4.73)	0.208	2.71(1.26-3.44)	2.94(2.18-4.11)	0.119
TNF-β	2.90(1.31-3.61)	11.66(8.84-15.46)	0.000^**^	2.64(1.18-3.60)	3.85(3.23-9.36)	0.000^**^

*P< 0.05, **P< 0.01.

**Figure 2:**
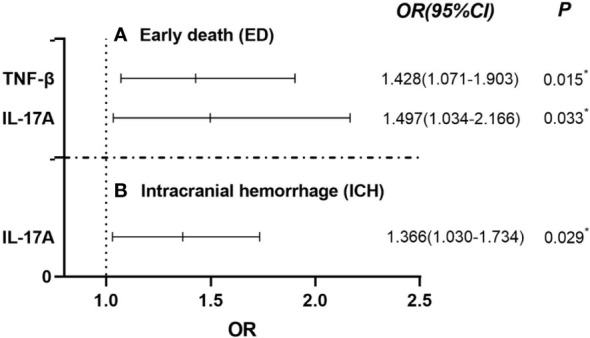
Using Binary Logistic Regression, statistically significant cytokines were screened out by univariate analysis and further multivariate analysis was used to test showed that TNF-β and IL-17A were independent risk factors for ED in APL patients [**(A)** Early death (ED)]. In addition, IL-17A was also an independent risk factors for ICH in APL [**(B)** Intracranial hemaorrhage (ICH)]. *P< 0.05.

The cytokines shown in **Table 5**, especially the statistically significant parameters, were included in the logistic regression univariate analysis of factors influencing ED in patients with APL, and the results were similar to those in **Table 5**. Further multivariate analysis suggested that two variables were independent risk factors for ED in newly diagnosed APL patients. These were TNF-β *OR* 1.428 (95% *CI*, 1.071 to 1.903) and IL-17A *OR* 1.497 (95% *CI*, 1.034 to 2.166) (*P*< 0.05) (**Figure 2A**). The influence of the cytokines on ICH was analyzed at the same time, and the results showed that only IL-17A (*OR* 1.366, 95% *CI*, 1.030 to 1.734) was an independent influencing factor for ICH (*P*< 0.05) (**Figure 2B**).

Differentiation syndrome (DS) was diagnosed according to the definition of Frankel, et al. (21), observing an incidence of 25.21% (29/115). The overexpression of the following cytokines at initial diagnosis also enhanced the occurrence of DS: IL-1β (2.09 *vs* 1.81 pg/ml), IL-4 (3.23 *vs* 2.11 pg/ml), IL-5 (3.08 *vs* 1.85 pg/ml), IL-10 (6.67 *vs* 4.35 pg/ml), IL-17A (3.97 *vs* 2.35 pg/ml), and TNF-β (3.85 *vs* 2.64 pg/ml) (*P*< 0.05) (**Table 5**).

### Determination of cutoff value

**Figure 3:**
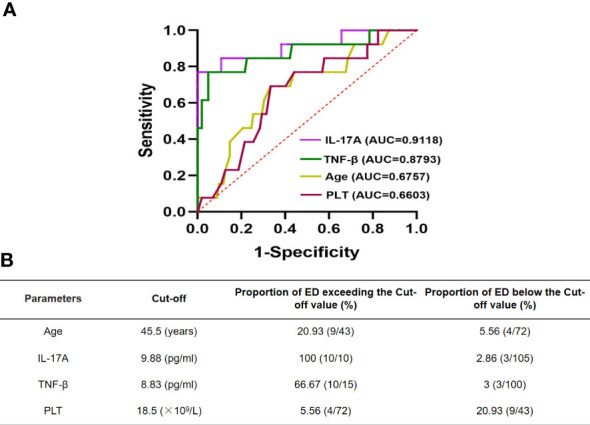
ROC curve of IL-17A, TNF-β and age in newly diagnosed APL patients with early death **(A)**. Cut-off values of them are determined by ROC curve **(B)**.

Cutoff values were calculated by the ROC curves to accurately determine the critical values of the parameters found to significantly affect ED in newly diagnosed APL patients. **Figure 3** shows a comparison of the area under the curves (AUCs) of IL-17A, TNF-β, age, and PLT between APL patients who experienced ED and survivors. AUCs of 91.18%, 87.93%, 67.57%, and 66.03% were found for IL-17A, TNF-β, age, and PLT, respectively (**Figure 3A**). In addition, the cutoff values were 9.88 pg/ml for IL-17A, 8.83 pg/ml for TNF-β, 45.5 years for age, and18.5×10^9^/L for PLT (**Figure 3B**).

## Discussion

As a unique subtype of AML, APL was previously considered one of the most rapidly lethal forms of AML. APL is characterized by the t(15;17)(q22; q12) chromosomal rearrangement, resulting in the formation of the fusion gene and protein PML/RARα. The three-year overall survival rates (three-year OS) of AML were reported to be 66% and 33% in patients younger and older than 60 years, respectively (22). However, with the use of ATRA and ATO, molecular-targeted agents that induce differentiation and apoptosis, in combination with chemotherapy when necessary, the complete response (CR) rates have increased to 90%-95%, and the five-year disease-free survival (five-year DFS) rates to over 90% for APL (3, 23, 24). Compared with the life-threatening initial stage of APL, patients who survive this critical period have better outcomes than those with other AML subtypes, resulting in a low risk of recurrence and a high survival rate, such that the disease is now highly curable. In the present study, except for 13 patients who experienced ED, the remaining 102 patients (88.79%, 102/115) achieved CR after induction therapy.

Prior to the ATRA era, early mortality was a serious problem, most of which could be attributed to bleeding complications (25). The detailed features of APL were first described by Bernard, et al. in 1959, who attributed the severe hemorrhagic diathesis to DIC or hyperfibrinolysis (26). However, despite the incorporation of ATRA and aggressive transfusion support in treatment, greatly improving the long-term prognosis of APL, early hemorrhagic death has remained significant (10, 27). Due to the complex coagulopathy associated with APL, intracerebral and pulmonary hemorrhages are the most common causes of death before and shortly after the initiation of treatment. Rodeghiero, et al. reported that the overall occurrence of early hemorrhagic death (within 0 to 10 days of treatment) was 9.4% in APL (28). In this study, we compared the clinical characteristics of APL with those of other AML subtypes. It was found that the EDR in APL was higher than in AML (non-APL) (11.30 *vs* 2.89%) and 11 out of 13 patients with ED died within a week of presentation. Furthermore, our study confirmed that APL patients had a higher incidence of bleeding and DIC than patients with other types of AML, together with increased rates of intracerebral hemorrhage. However, of the APL patients who showed bleeding at diagnosis, only one had pulmonary hemorrhage combined with cerebral hemorrhage (1/115 0.87%), in contrast to the findings of previous reports (2/732 0.27%, 1/34 2.95%, respectively) (29, 30). Due to the low number of cases where these two forms of lethal hemorrhage were combined, the significance of these differences in incidence could not be determined. Furthermore, the present study found that ICH was an independent risk factor for ED in newly diagnosed APL patients. The ICH-induced EDR in newly diagnosed APL patients was 6.96% (8/115), accounting for a high proportion of ED in the deceased patients (8/13, 61.54%), demonstrating that hemorrhage was still the most common cause of ED. This result is consistent with the findings of previous reports (31). Additionally, the median age at the time of diagnosis in APL was 38 years which was significantly lower than the median age of 53 years for patients with other types of AML. Likewise, it was confirmed that early mortality from APL increased with age. Studies have shown that the onset of AML was related to chromosomal abnormalities and genetic mutations (32–34) with some AML patients diagnosed with one or even multiple chromosomal abnormalities. The probability of chromosomal variation increases with age, which might be the reason why the median age of onset of AML patients (non-APL) was higher than that of APL patients.

As APL is now highly curable and has a younger age of onset, the identification of possible risk factors for ED and coagulopathy in APL patients is extremely important for determining an overall management strategy for the disease. It has been reported that ED in APL patients is associated with high WBC counts, ECOG scores, levels of lactate dehydrogenase, creatinine, and C-reactive protein, as well as DS and low PLT counts (8, 35, 36). The incidence of DS was found to be 25.21% in this study, which is consistent with the results described in a previous report (37). The pathogenesis of DS is related to both changes in serum cytokine levels (38) and cytokine storms (16). The adhesion of APL cells to the vascular wall is a key step in DS development (39). Risk stratification of APL affected long-term survival of APL patients (20, 40). In the present study, the high-risk group showed higher early mortality than the low/intermediate-risk group, consistent with a previous report (40). It has previously been suggested that the release of various cytokines, such as IL-1 and TNF-α, from leukemic promyelocytes might mediate coagulopathy in APL patients (41, 42). An *in vitro* study demonstrated that NB4 and HL-60 cells treated with ATRA and/or ATO gradually released the proinflammatory cytokines IL-1β and TNF-α (43). Secretion of IL-1β, IL-6, IL-8, and TNF-α by APL cells undergoing differentiation by ATRA was reported in a review (44). ATRA-induced therapy could result in the release of cytokines and adhesion molecules that promote the adhesion and aggregation of leukemic promyelocytes (15, 45). Furthermore, the adhesion of APL cells to endothelial cells (ECs) has been found to activate localized clotting and thrombus formation due to the release of cytokines and proangiogenic factors, leading to coagulopathy and early hemorrhagic death in APL patients (39, 45). The NCCN guidelines recommended the treatment of high-risk APL patients with corticosteroids, which can correct existing or worsening coagulopathy (17). Prophylactic corticosteroids were recommended for high-risk APL patients at presentation or for patients with increased WBC after ATRA initiation (46). In addition, in clinical work, we also found that the use of glucocorticoids to control cytokine release in the initial stage of the disease could mitigate abnormal coagulation. Therefore, in this study, we compared the levels of various cytokines between patients with APL and those with other AML types, and analyzed the effects of cytokines on ICH and ED in APL. The results showed that cytokine levels in APL were generally higher than in other types of AML, except for IL-2, IL-4, and TNF-α, with the TNF-α result differing from the findings of the previous report. Other AML subtypes are associated with increased TNF-α, including M4 and M5 (47), which might account for the lack of difference in TNF-α levels between APL and non-APL AML. Furthermore, in APL patients, cytokine levels were generally higher in the ED group than in the control group. These findings suggest that compared with other patients with AML, APL patients are in a hyperinflammatory state, which may affect the coagulation function of these patients and further contribute to early hemorrhagic death.

Although bleeding is the primary manifestation of APL-associated coagulopathy, patients are also at risk for thrombosis. Thrombosis occurred in 9.6-12% of APL patients and 3.2% of non-APL patients with AML at diagnosis (30, 48). The pathophysiological basis of DIC was the formation of diffuse microvascular thrombosis. More than 80% of patients with APL showed DIC-like coagulation abnormalities at presentation or after initiation of remission induction (49, 50). In this study, at presentation, DIC was diagnosed in 82.61% of APL patients, compared with an incidence of 16.01% in patients with other forms of AML. The fact that APL patients may have both bleeding and thrombosis underscores the complexity of coagulopathy pathogenesis and the variability of its manifestation. The pathophysiology of APL-associated coagulopathy is known to be complex (51) and has been associated with thrombin-activated DIC and annexin II-mediated primary hyperfibrinolysis, with hyperfibrinolysis, in particular, playing an important role in hemostatic derangement in APL (52, 53). In this study, it was found that plasma levels of FDP and D-Di were significantly increased in almost all newly diagnosed APL patients apart from one, suggesting that these patients were in a state of fibrinolytic activation. However, a large retrospective study reported that antifibrinolytic therapy alone had no advantage in improving early hemorrhagic death in APL patients (28). In addition, DIC is a complex process characterized by the initiation of coagulation and fibrinolytic activation, involving elevated levels of multiple procoagulant substances and plasminogen activators, such as tissue factor (TF), cancer procoagulant (CP), urokinase-type plasminogen activator (u-PA), and tissue-type plasminogen activator (t-PA), amongst others (54–58), resulting in the prolongation of PT and APTT and reducing the platelet counts. The initiation of DIC has traditionally been associated with extensive damage to the microvascular endothelium caused by localized or generalized inflammation in response to the release of host proteases, cytokines, and hormones (18). It was reported that patients severely ill with COVID-19 presented with a DIC-like coagulopathy (59) and it was suggested that the underlying mechanisms of COVID-19-associated coagulopathy might be associated with dysregulated immune responses orchestrated by inflammatory cytokines, lymphocyte cell death, hypoxia, and endothelial damage (60). Animal studies have shown that parasitic infections increased the levels of pro-inflammatory cytokines (TNF-α, IFN-γ, and IL-6) and induced significant changes in the hemostatic system, suggesting a correlation between inflammation and clotting (61). A variety of cytokines secreted from mononuclear, endothelial, and leukemic cells, including IL-1β, TNF-α, IL-8, and IL-6, promote hypercoagulability by upregulating the expression of procoagulant factors such as TF and adhesive molecules (AM) and downregulation of fibrinolytic factors such as thrombomodulin (TM) (38, 62, 63). Additionally, various cytokines released by leukemic promyelocytes, including IL-1β and TNF-α, have been shown to increase apoptosis and induce endothelial cell death, with a concomitant increase in TF expression (64). Both leukemic cells isolated from APL patients and NB4 cells have been found to express procoagulants including TF and CP (65, 66). The formation of the activated TF-VII complex led to intracellular signaling *via* protease-activated receptors to promote the transcription of cytokine genes and expression of procoagulant factors initiated the activation of the coagulation system. These reports together with the findings of the present study suggest that inflammatory cytokines play a role in coagulation dysfunction. APL is known to be combined with hyperinflammation and coagulopathy. To identify which cytokines were most important in coagulopathy in newly diagnosed APL and leading to the early death of these patients, we performed multivariate analysis, finding that IL-17A and TNF-β played significant roles in ED, especially IL-17A, which was also found to be an independent risk factor for ICH.

T helper 17 (Th17) cells, a novel subset of CD4^+^ T cells, are known to secrete IL-17A, IL-17F, IL-22, GM-CSF, and IFN-γ, which are involved in inflammation, autoimmune diseases, and graft-versus-host disease (GVHD) (67–72). As a signature cytokine of Th17 cell, IL-17A regulates the production of other lineage-specific cytokines by Th17 cells (73). The proportion of Th17 cells in peripheral blood mononuclear cells (PBMCs) from untreated patients with newly diagnosed AML have been shown to be significantly increased in comparison with those from healthy volunteers (74). Inflammation is closely related to coagulopathy, and TF acts as a bridge between endothelial activation, clotting, and inflammation (75). IL-17A had been reported to be involved in vascular inflammation (76), induce endothelial cell activation (77), and further up-regulate endothelial TF expression in liver cirrhosis (78). As an initiator of the exogenous coagulation pathway, the presence of TF in circulating blood activates the coagulation cascade, ultimately leading to thrombus formation (66). Nevertheless, it is not clear what role IL-17A plays in APL coagulation dysfunction. In this study, the coagulation function in APL patients was found to be worse than in patients with other types of AML and to be mainly manifested as prolonged PT, which reflects the exogenous coagulation pathway and is valuable in the prediction of bleeding. However, the APTT values in both groups of patients were within the normal reference range. A report showed that patients with PT ≥ 5 s had a relative risk of 6.14 for bleeding (14). Therefore, it is speculated that IL-17A may damage endothelial cells and induce overexpression of TF in newly diagnosed APL patients, which initiates the coagulation cascade and promotes the formation of generalized microthrombus, leading to hemorrhage secondary to the massive consumption of coagulation factors. This hypothesis will provide a new means of intervention to reduce the bleeding leading to early death in APL patients in the future. Of course, further research is needed to confirm this idea, and the molecular mechanism by which IL-17A regulates TF expression requires further investigation.

Taken together, although it is well-known that certain cytokines influence ED in newly diagnosed APL, there have been no reports on the cutoff values of IL-17A and TNF-β in the prediction of ED in these patients. This research clarifies the critical values of IL-17A and TNF-β that can be used to forecast ED, providing a new therapeutic strategy for reducing the early mortality of newly diagnosed APL patients. Aging and thrombocytopenia are also important causes of ED in these cases. The cutoff values were determined to be 45.5 years for age and 18.8×10^9^/L for PLT).

There are some limitations to this single-center retrospective study. First, the enrolled participants were all adults. The lack of data on pediatric patients restricts the generalization of the findings. Second, the mechanism by which IL-17A regulates coagulation and its effect on thrombocytopenia is not clear. Our further research will focus on these deficiencies to explore IL-17A as a predictor of early hemorrhagic death in newly diagnosed APL patients and its mechanism.

## Conclusions

In summary, our data indicated that the incidence of ED and DIC in newly diagnosed APL patients was higher than that in patients with other types of AML and, furthermore, APL patients have a younger age of onset than those with AML. ICH, age, and PLT at diagnosis were found by multivariate analysis to be independent factors predicting ED in newly diagnosed APL patients, especially ICH. In addition, the levels of cytokines at the initial APL diagnosis were shown to be generally higher than those in AML (non-APL) patients. Increased levels of IL-1β, IL-10, IL-17A, IL-17F, and TNF-β can cause the prolongation of PT in APL patients. In addition, the multivariate analysis showed that the elevated levels of IL-17A and TNF-β were directly related to ED in newly diagnosed APL patients, particularly IL-17A which was also found to affect ICH in APL patients. To sum up, age, PLT counts, and the levels of cytokines IL-17A and TNF-β influence ED in newly diagnosed APL patients. Moreover, ICH is the main contributor to ED in *de novo* APL patients. The levels of cytokines IL-17A and TNF-β were strongly correlated with ED in APL, particularly IL-17A, which was found to be an independent risk factor for ICH. Therefore, it is suggested that increased production of IL-17A leads to an increased risk of early hemorrhagic death in newly diagnosed APL patients by inducing TF overexpression to initiate coagulation and further leading to the excessive depletion of clotting factors. Furthermore, IL-17A may be considered a predictive cytokine for ED in newly diagnosed APL patients.

## Data availability statement

The original contributions presented in the study are included in the article/**Supplementary Material**. Further inquiries can be directed to the corresponding author.

## Ethics statement

The studies involving human participants were reviewed and approved by Ethics Committee of the First People’s Hospital of Yunnan Province. The patients/participants provided their written informed consent to participate in this study.

## Author contributions

SZ analyzed the data and drafted the manuscript. YG reviewed the analysis results and graphs. ZL performed statistics. TY designed this plan, reviewed the manuscript and revised the main content. And all authors contributed to the article and approved the submitted version.
