# Supplementary figures and images for: Influence of cytokines on early death and coagulopathy in newly diagnosed patients with acute promyelocytic leukemia
Source: Front Immunol. 2023 Mar 31;14:1100151. doi: 10.3389/fimmu.2023.1100151 (PMC10103902; doi:10.3389/fimmu.2023.1100151)

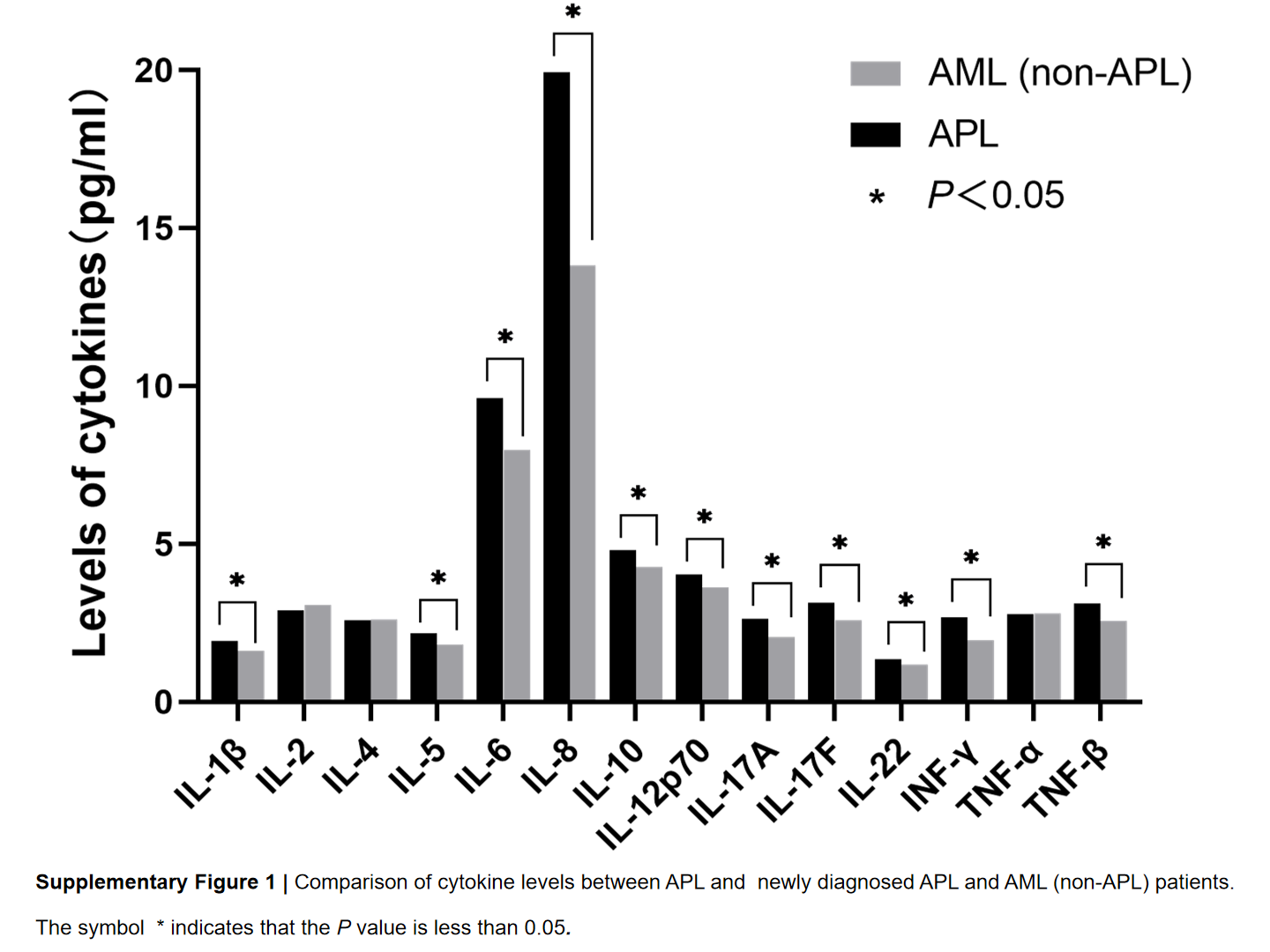

Supplement: Supplementary file 1 [file Image_1.tif]

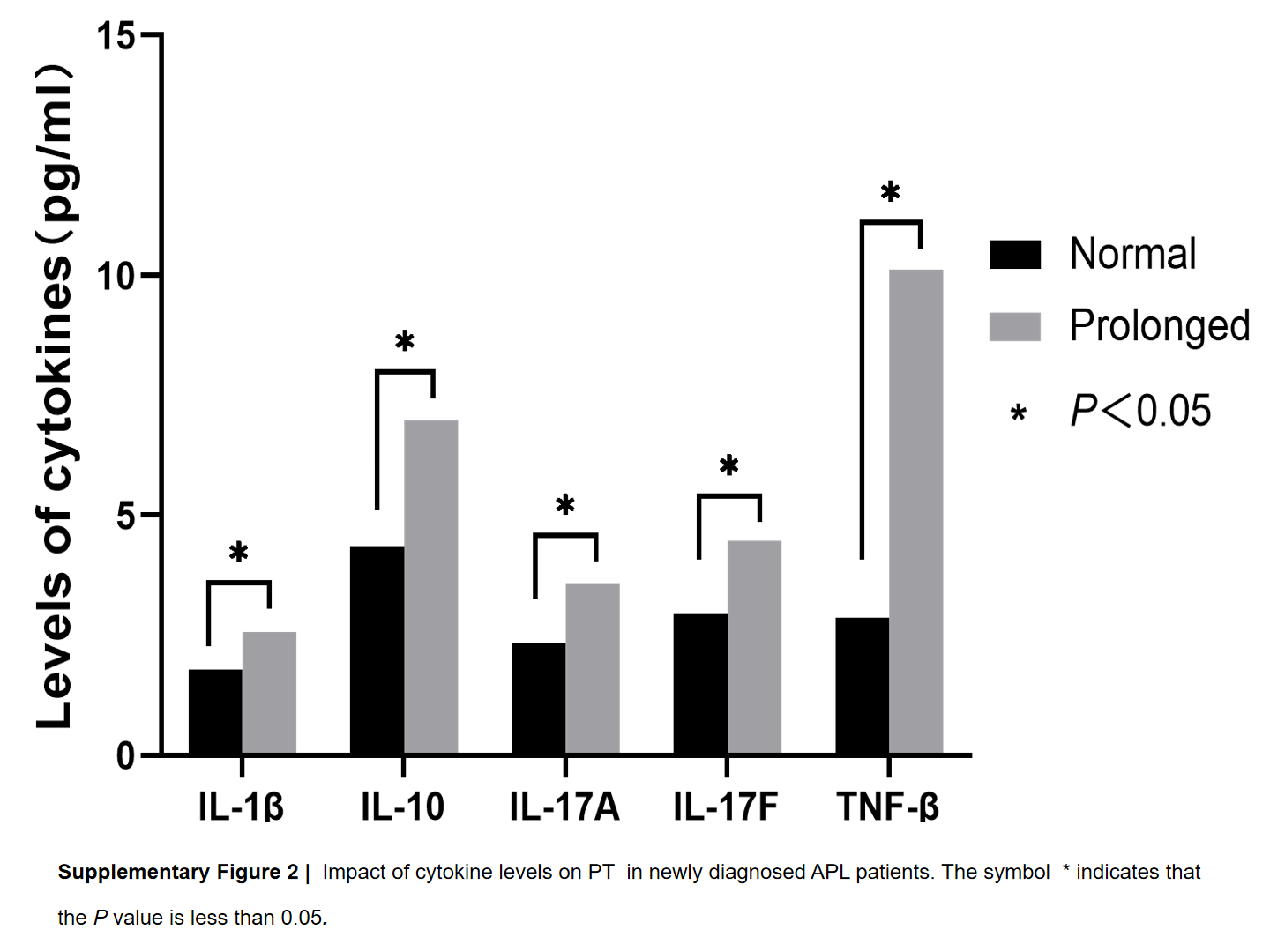

Supplement: Supplementary file 2 [file Image_2.tif]

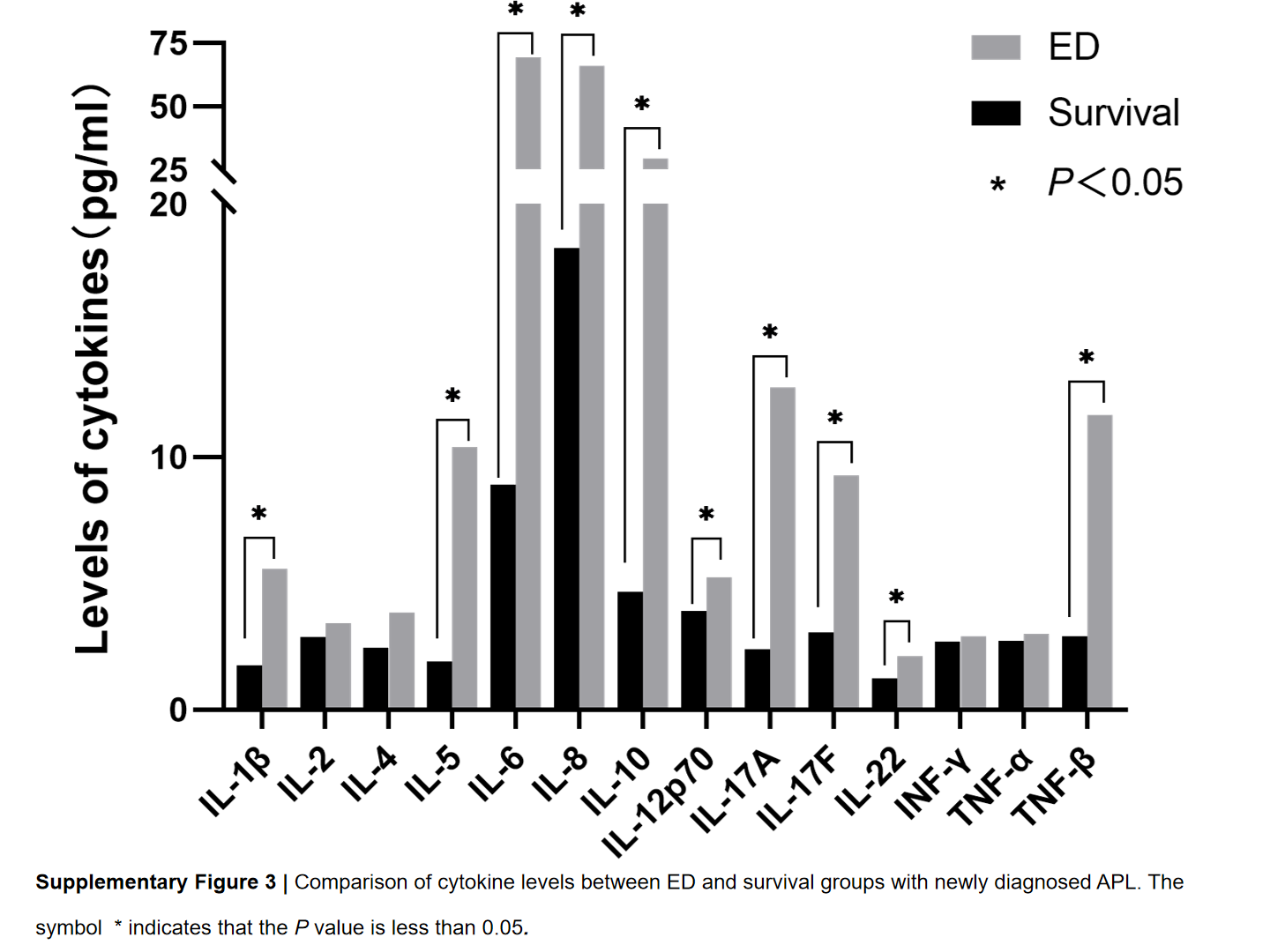

Supplement: Supplementary file 3 [file Image_3.tif]
